# Deciphering Transcriptome and Complex Alternative Splicing Transcripts in Mammary Gland Tissues from Cows Naturally Infected with *Staphylococcus aureus* Mastitis

**DOI:** 10.1371/journal.pone.0159719

**Published:** 2016-07-26

**Authors:** Xiu Ge Wang, Zhi Hua Ju, Ming Hai Hou, Qiang Jiang, Chun Hong Yang, Yan Zhang, Yan Sun, Rong Ling Li, Chang Fa Wang, Ji Feng Zhong, Jin Ming Huang

**Affiliations:** Dairy Cattle Research Center, Shandong Academy of Agricultural Sciences, Jinan, Shandong, P.R. China; Wageningen UR Livestock Research, NETHERLANDS

## Abstract

Alternative splicing (AS) contributes to the complexity of the mammalian proteome and plays an important role in diseases, including infectious diseases. The differential AS patterns of these transcript sequences between the healthy (HS3A) and mastitic (HS8A) cows naturally infected by *Staphylococcus aureus* were compared to understand the molecular mechanisms underlying mastitis resistance and susceptibility. In this study, using the Illumina paired-end RNA sequencing method, 1352 differentially expressed genes (DEGs) with higher than twofold changes were found in the HS3A and HS8A mammary gland tissues. Gene ontology and KEGG pathway analyses revealed that the cytokine–cytokine receptor interaction pathway is the most significantly enriched pathway. Approximately 16k annotated unigenes were respectively identified in two libraries, based on the bovine Bos taurus UMD3.1 sequence assembly and search. A total of 52.62% and 51.24% annotated unigenes were alternatively spliced in term of exon skipping, intron retention, alternative 5′ splicing and alternative 3ʹ splicing. Additionally, 1,317 AS unigenes were HS3A-specific, whereas 1,093 AS unigenes were HS8A-specific. Some immune-related genes, such as ITGB6, MYD88, ADA, ACKR1, and TNFRSF1B, and their potential relationships with mastitis were highlighted. From Chromosome 2, 4, 6, 7, 10, 13, 14, 17, and 20, 3.66% (HS3A) and 5.4% (HS8A) novel transcripts, which harbor known quantitative trait locus associated with clinical mastitis, were identified. Many DEGs in the healthy and mastitic mammary glands are involved in immune, defense, and inflammation responses. These DEGs, which exhibit diverse and specific splicing patterns and events, can endow dairy cattle with the potential complex genetic resistance against mastitis.

## Introduction

Bovine mastitis is an inflammation of the mammary gland invaded and infected by bacteria. This disease results in considerable economic loss and engenders food safety and animal welfare concerns in the dairy industry [1]. The major microorganisms responsible for mastitis are *Staphylococcus aureus* (*Staph*. *aureus*), *Streptococcus* and *Escherichia coli* (*E*. *coli*). Among these three pathogens, *Staph*. *aureus* is the most frequent cause of udder infection [2]. Obtaining insights into the processes of bovine defense and immune response to mastitis could provide new solutions to mastitis infection. Moreover, a genetic strategy based on the molecular mechanism of cow mastitis demonstrates positive effects on the reduction of antibiotic use in dairy cow breeding and improves the safety of milk products [3].

The alternative splicing (AS) of genes is a common phenomenon in mammalian tissues and cell types in response to stimulations in the eukaryon [4–6]. A gene can produce multiple mRNA transcripts and diverse protein isoforms through this process; subsequently, the gene differentiates proteins to display various binding properties, intercellular localizations, and expression regulations, resulting in related, distinct, or even opposing functions [7–9]. Next-generation sequencing technology is a rapid and cost-effective approach to screen functional candidate genes, differentially expressed genes (DEGs), and important signal pathways that preliminarily explain the molecular mechanism in various tissues. This technology is also used to further identify gene AS related to important economic traits of interest [10,11]. Recent genome-wide association studies have reported that approximately 27.22% of genes in the bovine embryo [12], 38.85% of genes in the adipose tissue [9], and >90% of genes in human tissues [13] undergo AS. More importantly, splice variants of many immune-related genes are associated with various diseases, such as bovine mastitis [14–16]. These variants are one of the major determinant factors of diseases [17]. Currently knowledge on the molecular mechanism underlying the individual differences in immune response to bovine mastitis, especially at the later stages of natural infection with pathogens, is still limited. The interaction between mastitis pathogens and the host immune system is extremely complex [18]. We hypothesize that the differences induced by the AS of genes can be used by the immune system of cows to process complex information to initiate host response to invading pathogens. Therefore, distinguishing the transcriptomic characteristics and differential patterns of AS in bovine mammary glands between healthy and mastitic cows naturally infected with *Staph*. *aureus* is important.

To investigate the relevant genes involved in bovine mastitis susceptibility and their regulatory patterns, we initially selected mammary glands from healthy and mastitis-infected cow groups to perform transcriptome sequencing using Illumina HiSeq^TM^ 2000 platform. We obtained a number of candidate genes and signal pathways related to inflammation, defense, and immune responses according to the gene functional annotation and comprehensive analysis of patterns of gene expression. Our findings can provide a foundation for further research on the specific functions of candidate genes related to bovine mastitis susceptibility. The results can also elucidate the molecular process and potential mechanism of cow response to natural infection with *Staph*. *aureus*.

## Materials and Methods

### Ethics Statement

All experiments were carried out according to the Regulations for the Administration of Affairs Concerning Experimental Animals published by the Ministry of Science and Technology, China in 2004 and approved by the Animal Care and Use Committee from the Dairy Cattle Research Center, Shandong Academy of Agricultural Sciences, Shandong, P. R. China.

### Mammary gland tissues and RNA isolation

Six 3- to 5-year-old Holstein cows were obtained from the standardized dairy farm of Shandong Province, China. Three of these cows were healthy, whereas the other three were mastitic cows infected with *Staph*. *aureus*. Briefly, a cow was defined as healthy if the clinical symptoms such as swelling, redness, hardness or pain were not observed in the udder and no main pathogens was examined from the cow’s mammary tissues using culture and PCR methods. The mastitis group used for this study was referred to as those cows with *Staph*. *aureus* and milk somatic cell count per mL above 1 million. At the slaughterhouse, a part of fresh mammary gland tissue were used for pathogen identification, another samples were cut, cleaned with RNase-free water, and then immediately frozen in liquid nitrogen until further use. After pathological evaluation, the three healthy and three mastitis-infected mammary glands were pooled as HS3A and HS8A groups, respectively. The total RNA from the two pool samples was extracted using Trizol reagent (Invitrogen) according to the manufacturer’s instructions. The quality of RNA samples was assessed with an Agilent 2100 Bioanalyzer (Agilent Technologies, Santa Clara, CA, USA). The RNA Integrity Number was 7.6–7.9, and the 28S/18S ratio was 1.9, and the OD260/280 ratio was 2.0.

### Construction of cDNA library and sequencing

The cDNA libraries of the two groups (HS3A and HS8A) were constructed as follows. First, total RNAs were isolated from the two samples, and the mRNAs were purified and enriched using magnetic beads with oligo (dT). Second, mRNA sequences were fragmented, and first-strand cDNAs were synthesized using the cleaved segments as a template with six base random primers (Illumina). Subsequently, second-strand cDNA synthesis was performed by adding the buffer, dNTPs, RNase H, and DNA polymerase I. Then, the synthesized cDNA was subjected to end-repair and phosphorylation using T4 DNA polymerase, Klenow DNA polymerase and T4 PNK. Third, Poly (A) and sequencing joints were added to the cDNA. Fourth, the products of ligation reaction were purified on a 2% TAE-agarose gel. A range of cDNA fragments (200 ± 25 bp) were selected from the agarose gel. Fifteen rounds of PCR amplification were performed to enrich the cDNA template using PCR Primer PE 1.0 and PE 2.0 (Illumina) with Phusion DNA Polymerase. Finally, the two constructed libraries were sequenced using the PE technology (2 × 75 bp read length) Illumina HiSeq^TM^ 2000 platform (BGI, Shenzhen, China). The randomness of mRNA fragmentation was evaluated with the distribution of reads in the reference genes (Fig A in S1 File).

### Data filtering and transcriptome assembly

Raw reads were cleaned by removing adapter sequences as well as reads with too many unknown base calls (N), low complexity, and low-quality bases, and data quality was controlled by a stringent process to improve the accuracy of the transcriptome analysis results. Reads were filtered following three criteria. (1) Reads with adapter contaminant were first removed. (2) Reads in which the percentage of unknown nucleotides was higher than 5%, the corresponding reads were discarded. (3) Reads in which the percentage of bases with a quality score of ≤5 was greater than 50% were eliminated. After clean data from the HS3A and HS8A were generated, transcriptome assemblies were performed using the SOAP2 software [19]. The genome assembly *Bos taurus* UMD3.1 deposited in Ensembl (http://www.ensembl.org/info/data/ftp/index.html) was used as the reference genome. We gathered the paired-end (PE) reads with one end mapped on the unique contig and the other end located in the gap region to further shorten the remaining gaps. We also performed local assembly with the unmapped end to fill in the small gaps within the scaffolds. Such sequences that contain the least “N” and do not extend on either end were defined as unigenes.

### Identification of differentially expressed genes

The expression levels of genes were calculated using the RPKM method to identify the DEGs between the healthy HS3A and mastitic HS8A groups. This method can eliminate the influence of deviations in transcript lengths and sequencing levels [20]. The corresponding formula is RPKM = (10^6^*C*)/(*NL*/10^3^), where *C* is the number of fragments aligned to the exons of the gene, *N* is the number of total fragments aligned to all the genes, and *L* is the base number of the gene CDS. In our analysis, the DEGs were screened using a false discovery rate threshold value of ≤0.001 and an absolute value of log_2_ ratio of ≥1. All fold changes of differentially expressed genes are log2 values.

### Enrichment analysis of differentially expressed genes

The potential functions of assembled DEGs were predicted and annotated against the Gene ontology (GO) and KEGG protein databases. The GO terms especially enriched in DEGs were defined using hypergeometric distribution testing and Bonferroni correction with p-value ≤0.05 as the threshold. For the KEGG protein database, detailed information about each gene can be shown in a signal pathway to reveal its molecular regulatory network and metabolic pathway. The enrichment of a pathway is obtained by multiple testing and is considered significant if q-value ≤ 0.05.

### Alternative splicing analysis of genes

We used SOAPsplice software with a default setting and RNA-Seq data to detect splice junctions and identify the potential AS patterns of genes (Fig B in S1 File). SOAPsplice uses a novel approach comprising the following steps: (1) identifying as many reasonable splice junction candidates as possible and (2) filtering the false positives with two effective filtering strategies [21]. First, junction sites, which provide information about boundaries and combinations of different exons in a transcript, are detected by SOAPsplice. Then, all junction sites of the same gene are used to distinguish the type of AS event. A brief introduction of the algorithms used to detect the four AS events in this study is provided in Fig C in S1 File.

### Prediction of novel transcripts

Transcripts with reads were assembled using Cufflink [22]. Reads that are continuous and overlapping according to the distribution of reads on the reference genome would form a transcriptional activity area (TAR). Different TARs were linked to form an assembled transcript (Fig D in S1 File). If the gene models were found in intergenic regions (200 bp away from upstream or downstream genes), the transcripts were regarded as candidates for novel transcripts.

## Results

### Illumina paired-end sequencing and transcriptome assembly

RNA was extracted from the healthy and *Staph*. *aureus*-infected mammary tissues to generate a comprehensive survey of genes associated with mastitis infection. Each sequencing feature obtained using Illumina PE sequencing technology yielded 2 × 75 bp independent reads from either end of a cDNA fragment. In the present study, a total of 80% and 78.65% sequencing reads in the HS3A and HS8A were mapped onto the reference *Bos taurus* genome assembly (UMD3.1), respectively. Finally, the read assembly yielded approximate 16k unigenes with an average length of 2k bp in two libraries (Table 1).

**Table 1:** Sequencing and assembly results of two libraries.

	Healthy group (H3A)	Mastitic group (H8A)
Total number of reads	53333334	49245176
Total mapped reads	42664111	38733278
Percentage (%) of mapped reads to genome	80.00	78.65
Total unigenes	16247	16246
Minimun length (bp) of unigene	147	147
Maximun length (bp) of unigene	22023	22023
Average length (bp) of unigene	2165	2168

Determining subtle transcriptomic changes in the alveolar mammary tissues can provide insights into molecular mechanisms underlying biological processes, such as immune and defense responses against mastitic infection. In this study, 1,352 DEGs were identified, including 602 up-regulated genes and 750 down-regulated genes, and found specific to the HS8A mammary gland tissue (Table A in S2 File). The top 100 DEGs from the aforementioned genes were selected by the reads per kilobase of the exon model per million mapped reads (RPKM) calculation and further ranked based on the expression fold change value [log_2_(HS8A/HS3A)] of the genes (Table B in S2 File). Out of the 100 top DEGs, 66% (66/100) genes were up-regulated in the HS8A, indicating that these genes play important roles bovine response to mastitis.

A total of 1,352 DEGs were matched and classified into three functional categories, namely, molecular function (MF), biological process (BP), and cellular component (CC), according to the GO classification system. Among these DEGs, 1,028 matched genes were involved in molecular functions and were clustered into 367 classifications. The top 10 significant enrichment GO terms, such as the protein binding, cytokine activity, receptor binding, chemokine receptor activity, and chemokine binding were also significantly enriched (Table A in S3 File). For the BP, the “response to stimulus” term (32.5%, 332 out of 1,023 DEGs) was the most significant enrichment (Table B in S3 File). Moreover, 124, 74, 17, and 55 DEGs were significantly enriched (p < 0.05) and respectively classified into the GO terms immune system process, defense response, regulation of cell migration, and regulation of immune system process. In addition, 38, 30, 28, 22, 69, and 6 DEGs were respectively classified into the GO terms response to wounding, immune response, response to bacterium, inflammatory response, programmed cell death, and innate immune response in spite of their non-significant enrichments (p > 0.05, Table B in S3 File). The 60 DEGs participating in the “immune system process,” “defense response,” “immune response,” and “inflammatory response” GO terms and expressing with above 1.5-fold change in two groups are listed in S4 File. For the CC analysis, “extracellular region” was the most abundant GO term (Table C in S3 File).

**Fig 1:**
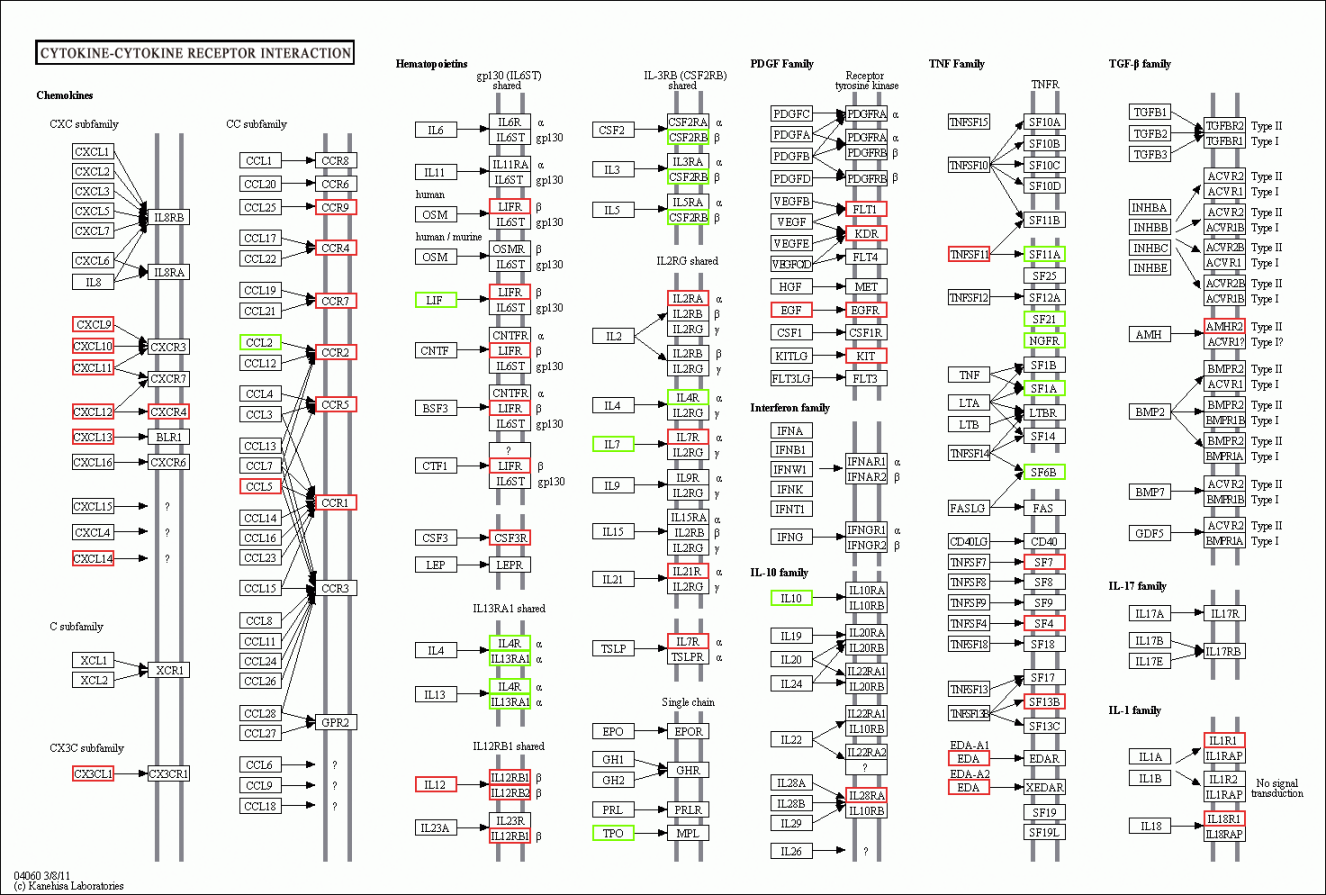
Differentially expressed genes participating in cytokine–cytokine receptor interaction pathway. Genes enclosed in red boxes are up-regulated, whereas those in green boxes are down-regulated in the mammary gland tissues of mastitis-infected with respect to those in the mammary gland tissues of healthy cows.

To further understand the biological functions of the unigenes, we mapped DEGs to the signal pathways described in KEGG. Consequently, 1,349 genes were assigned to the KEGG database, and these genes were classified into 221 biological pathways based on the reference canonical pathways. Thirty-three highly ranked KEGG pathway were significantly enriched (p<0.05,Table D in S3 File) and seven pathways are associated with immune, such as the “cytokine–cytokine receptor interaction” pathway (q< 0.05) is the most significant pathway with immunological function (Fig 1). These related genes could act as important regulatory knots during mastitis infection.

### Novel transcripts and mastitis-related quantitative trait locus (QTLs)

**Fig 2:**
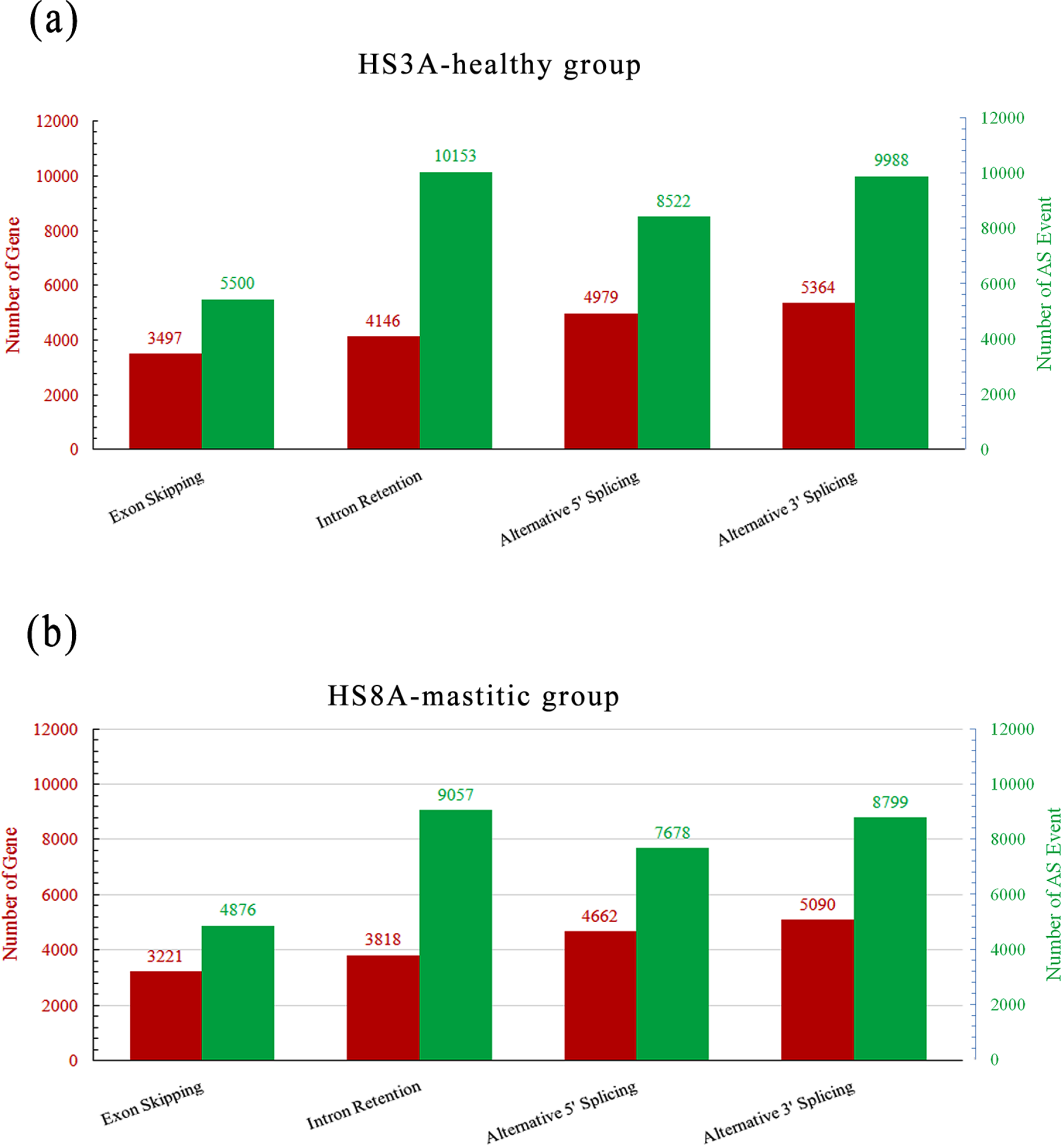
Novel transcripts harboring QTLs associated with clinical mastitis in the two groups. BTA: Bovine autosome.

We identified new transcripts because of the imperfection of the current annotation of the available transcriptome database. A sum of 118,045 and 108,279 candidates for novel transcripts from the 29 autosomes, as well as chromosome MT and X, were identified in the healthy and mastitic tissues, respectively. The transcripts of the mastitic group had an average length of 304 bp, 5 reads per bp, and 2 exons. Similarly, the transcripts of the healthy group possessed approximately 306 bp length, 5 reads per bp, and 2 exons. Using the location of the transcripts in the AnimalQTL (thttp://www.animalgenome.org/cgi-bin/QTLdb/BT/search), we found 4,321 (3.66% of total novel transcripts) novel transcripts of the healthy group and 5,848 (5.4%) novel transcripts of the mastitic group from BTA2, 4, 6, 7, 10, 13, 14, 17, and 20, which were located in the QTLs involved in clinical mastitis (Fig 2; S5 File). A total of 154 candidate novel transcripts (TU45009–TU45164) were specific to the mastitic group (Table B in S5 File). These transcripts were located in the BTA2 (45.57–50.89 Mb), harboring the QTL (46.2–53.0 Mbp) associated with clinical mastitis in Norwegian Red cattle [23] and *Staph*. *aureus* incidence rate of clinical mastitis in Holstein cows [24]. Another HS8A-specific novel transcript harbored the QTL associated with the duration of mastitis in Polish Holstein–Friesian cows [25]. Specific novel transcripts were identified in the same chromosomes and QTL regions in the two groups. For instance, 677 novel transcripts expressed only in the mastitic group were located in the region BTA14:1.89–16.51 Mb, harboring the QTL that affects somatic cell score and mastitis in Finnish Ayrshire cattle [26]. Moreover, a few transcripts, such as TU23644 and TU25507, were common in the two groups (Tables A and B in S5 File). The results suggest that mastitis-specific novel transcripts are candidate novel genes for mastitis susceptibility in cows.

### Alternative splicing analysis between healthy and mastitic bovine mammary gland tissues

**Fig 3:**
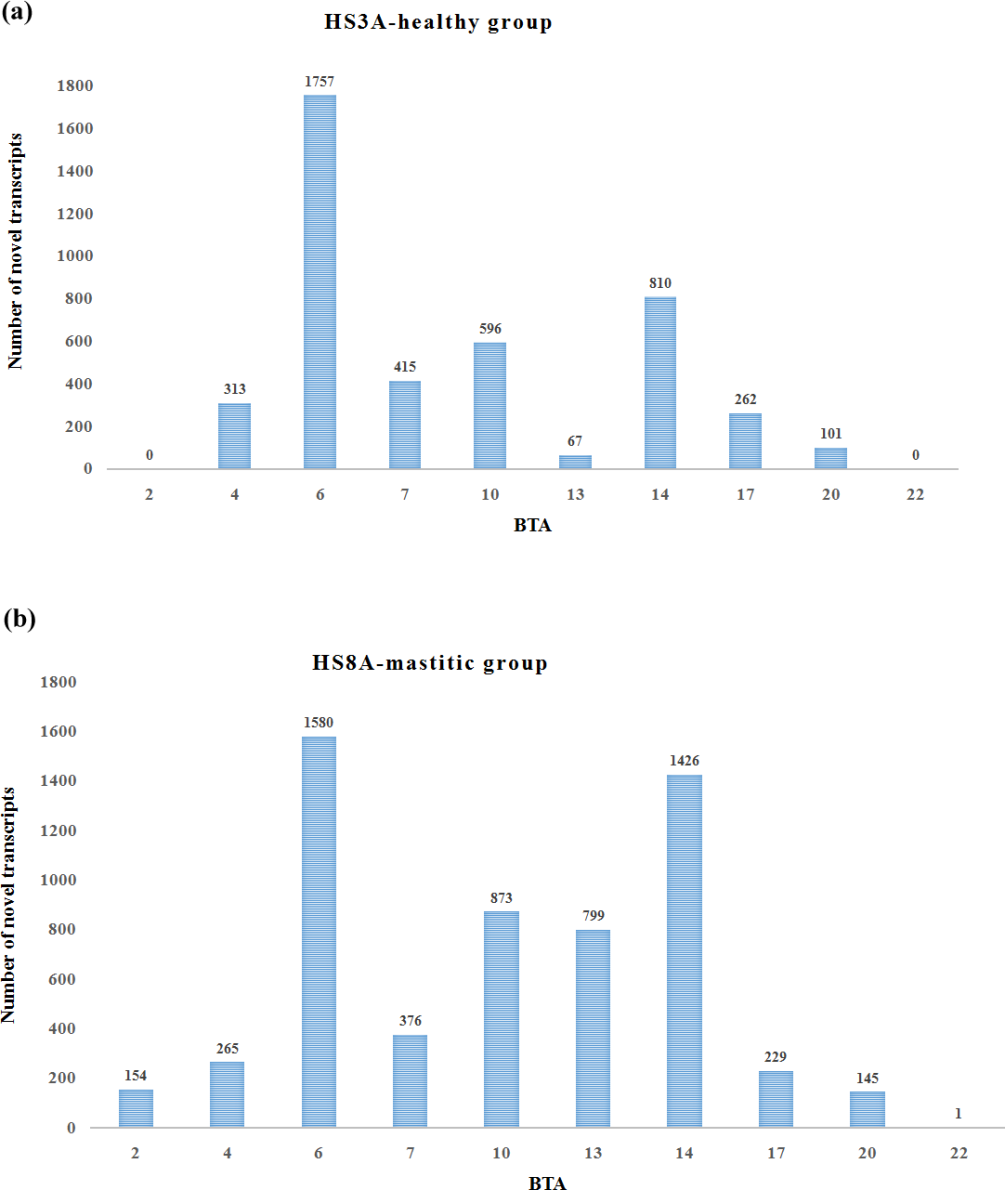
Genes and their patterns of alternative splicing events in healthy and mastitic cow’s mammary gland tissue. The red and green histograms show the numbers of genes and the corresponding numbers of alternative splicing events, respectively.

Seven patterns of gene AS include exon skipping, intron retention, alternative 5′ splice site, alternative 3′ splice site, alternative first exon, alternative last exon, and mutually exclusive exon [7], as shown in the Fig A in S1 File. In the present study, four patterns of gene AS, namely, exon skipping, intron retention, alternative 5′ splice site, and alternative 3′ splice site in the mammary gland transcriptome were detected using the SOAP splice program (Fig B in S1 File). A total of 8,549 (52.62%) and 8,325 (51.24%) unigenes were alternatively spliced, exhibiting 34,523 and 30,410 AS events (Fig 3). In total, 1,317 alternatively spliced unigenes were HS3A-specific, whereas 1,093 alternatively spliced unigenes were HS8A-specific. Further classification of these AS events reveal that a total of 3,497 and 3,221 unigenes demonstrated exon skipping in HS3A and HS8A, respectively. Moreover, 4,146 and 3,818 unigenes presented intron retention, 4,979 and 4,662 unigenes displayed alternative 5′ splicing, 5,364 and 5,090 unigenes showed alternative 3′ splicing in HS3A and HS8A, respectively (Fig 3).

The DAVID functional clustering analysis showed that 51 co-expressed genes that participate in the immune, defense, and inflammatory responses showed intron retention (Table A in S6 File). Of these genes, 12 genes (MYD88, C2, TFE3, VDAC1, ENPP2, TLR6, OAS2, IL27R, CD163, CD8A, ENPP1, and SAA3) and eight genes (NCF1, ADA, ITGB6, MAP2K3, MLF2, CSF3, POLR3C, and TNFRSF1B) were healthy-group-specific and mastitic-group-specific, respectively (Table 2). The number of intron retention events of each gene ranged from 1 to 14. For example, the alpha-S2-casein Casocidin-1 (ENSBTAG00000005005) gene has the highest number (14) of intron retention events. Alpha-S2-casein Casocidin-1 is an antibacterial peptide that exhibits antibacterial activity and inhibits the growth of *E*. *coli* and *Staph*. *aureus* [27]. These genes and their variants probably play important roles in mastitis susceptibility or resistance in dairy cattle.

**Table 2:** Genes with retained intron events were health-specific and mastitis-specific and participated in immune, defense and inflammatory responses.

Gene ID	Gene name	Chromosome	Retained intron	Intron
Healthy group-specific				
ENSBTAG00000000563	myeloid differentiation primary response gene 88	Chr22	11727419–11728296	intron 1
ENSBTAG00000007450	complement factor B	Chr23	27208004–27208102	intron 15
ENSBTAG00000008523	transcription factor binding to IGHM enhancer 3	ChrX	55404582–55404656	intron 4
ENSBTAG00000013113	voltage-dependent anion channel 1	Chr7	44848653–44850305	intron 7
ENSBTAG00000013165	ectonucleotide pyrophosphatase/phosphodiesterase 2	Chr14	80000212–80001328	intron 9
ENSBTAG00000014031	toll-like receptor 1; toll-like receptor 6	Chr6	60361050–60363020	intron 2
ENSBTAG00000014628	2'-5'-oligoadenylate synthetase 2, 69/71kDa; 2',5'-oligoadenylate synthetase 1, 40/46kDa	Chr17	64576616–64577474	intron 4
ENSBTAG00000016933	interleukin 27 receptor, alpha	Chr7	9861570–9861654	intron 4
ENSBTAG00000019669	CD163 molecule	Chr5	109203830–109205560	intron 14
ENSBTAG00000021141	CD8a molecule	Chr11	49852583–49853129	intron 2
ENSBTAG00000021830	ectonucleotide pyrophosphatase/phosphodiesterase 1	Chr9	71911345–71912280	intron 16
ENSBTAG00000022396	serum amyloid A 3	Chr29	27687476–27688031	intron 1
Mastitis group-specific				
ENSBTAG00000003305	neutrophil cytosolic factor 1	Chr25	34809853–34810612	intron 6
ENSBTAG00000005280	adenosine deaminase	Chr13	73796362–73797546	intron 5
ENSBTAG00000009080	integrin, beta 6	Chr2	37481846–37488767	intron 15
ENSBTAG00000010576	mitogen-activated protein kinase kinase 3	Chr19	36313234–36313726	intron 11
ENSBTAG00000014931	myeloid leukemia factor 2	Chr5	10677366–10677618	intron 2
ENSBTAG00000021462	colony stimulating factor 3 (granulocyte)	Chr19	41597296–41597520	intron 4
ENSBTAG00000021637	polymerase (RNA) III (DNA directed) polypeptide C (62kD)	Chr3	23394542–23394652	intron 12
ENSBTAG00000024928	tumor necrosis factor receptor superfamily, member 1B	Chr16	38246513–38252554	intron 8

In addition to genes with retained introns, 21 genes involved in the immune, defense, and inflammatory responses were enriched and displayed exon skipping in both the healthy and mastitic groups (Table B in S6 File). Out of these genes, six genes (CD4, CCL28, C1S, CFB, IL1R1, and MIF) were specific to the healthy group and six genes (ADA, CCL5, COLEC12, CYBA, POLM, and THBS1) were specific to the mastitic group, respectively (Table 3). In addition, four genes (BOLA, SWAP70, NFKB2, and TLR4) were co-expressed; and they exhibited the same exon skipping events in the HS3A and HS8A groups. Five genes (CSN1S2, LTF, TMEM173, PSMB8, and CD46) displayed different exon-skipping patterns, generating distinct transcripts (Table B in S6 File). For example, eight exon skipping events of the bovine LTF gene were detected in the healthy group, whereas five exon skipping events were detected in the mastitic group.

**Table 3:** Genes with exon skipping events were health-specific and mastitis-specific and participated in immune, defense and inflammatory responses.

Gene ID	Gene name	Chromosome	Strand	Constitutive exon	Inclusive exon	Constitutive exon
**Healthy group-specific**						
ENSBTAG00000003255	CD4 molecule	Chr5	-	10617626–10617693	10618041–10618162	10618885–10619085
ENSBTAG00000001557	chemokine (C-C motif) ligand 28	Chr20	+	33364491–33364589	33379174–33379300	33382728–33382963
ENSBTAG00000004840	complement component 1, s subcomponent	Chr5	-	10394917–10395124	10395366–10395491	10396391–10396555
ENSBTAG00000007450	complement factor B	Chr23	-	27220514–27220652	27221729–27221862	27222058–27222159
ENSBTAG00000005273	interleukin 1 receptor, type I	Chr11	+	7125679–7125830	7131304–7131447	7132681–7132848
ENSBTAG00000007375	macrophage migration inhibitory factor (glycosylation-inhibiting factor)	Chr17	+	74576931–74577135	74577275–74577447	74577530–74577723
**Mastitic group-Specific**						
ENSBTAG00000005280	adenosine deaminase	Chr13	-	73793223–73793352	73794689–73794753	73795053–73795154
ENSBTAG00000007191	chemokine (C-C motif) ligand 5	Chr19	+	13970861–13970997	13972504–13972615	13977129–13977615
ENSBTAG00000007705	collectin sub-family member 12	Chr24	-	36386236–36386724	36400382–36401428,36402633–36402731	36412276–36412398
ENSBTAG00000003895	cytochrome b-245, alpha polypeptide	Chr18	-	12882129–12882210	12882703–12882786	12883011–12883085
ENSBTAG00000007796	polymerase (DNA directed), mu	Chr4	+	79977189–79977394	79978982–79979165	79979695–79979793
ENSBTAG00000002006	thrombospondin 1	Chr10	+	35120858–35121031	35121264–35121391	35121495–35121647

In the healthy and mastitic groups, 17 genes (BMPR1A, IL18, SMAD1, CD4, NCF1, TNFRSF1A, CD46, CCL25, TLR4, CARD9, S1PR3, ENPP2, PSEN2, MLF2, LYST, SAMHD1, and SAA3) and 9 genes (PNLIPRP2, NLRP3, IL1R-I, ITIH4, C7, COTL1, HIF1A, IL17D, and C4A) that were involved in immune, defense, and inflammation responses underwent alternative 5′ splicing site pattern (Table C in S6 File). Eight genes shared the same alternative 5′ splicing events, whereas 17 genes were expressed in different alternative 5ʹ splicing events (Table D in S6 File).

Twenty genes displayed healthy-group-specific alternative 3′-splicing events, whereas nineteen genes expressed mastitic-group-specific alternative 3′-splicing site patterns (Table E in S6 File). Between the two groups, 26 genes shared the same AS patterns, whereas 17 genes had different AS patterns (Table F in S6 File).

In total, 109 genes that are associated with immune, inflammation and defense were alternatively spliced. Further, we found eight genes involved in immune, inflammation and defense were differentially expressed (≥1.5-fold change) and alternatively spliced in the healthy and mastitic tissues, such as C7, CCL19, CCL5, CD4, CSN1S2, ACKR1, ITIH4 and PNLIPRP2 genes (Table E in S4 File).

## Discussion

In the present study, we performed transcriptome sequencing of mammary glands from healthy cows and mastitis-infected cows, for which we respectively generated comprehensive transcriptome data of approximately 30 million and 29 million clean reads. We analyzed and identified 1,352 DEGs and complex AS patterns of genes between the healthy and mastitic tissues. Many of the genes have various transcripts involved in the inflammation, defense, and immune processes against infection. We also investigated the potential relationships between candidate novel transcripts and known QTLs associated with clinical bovine mastitis.

Using the clustering analyses of GO protein databases for DEGs, we identified a considerable number functional molecular processes related to the immune defense system, such as the immune system process, defense response, immune response, and inflammatory response terms. Among these terms, the immune system process and defense response terms were significantly enriched. We performed KEGG pathway analysis and found that several important pathways, such as cytokine–cytokine receptor interaction (64 DEGs), chemokine signaling pathway (53 DEGs), and complement and coagulation cascade pathways (32 DEGs), were also enriched. The aforementioned pathways are related to immunological functions. Interestingly, the cytokine–cytokine receptor interaction pathway is only a part of the chemokine signaling pathway. Moreover, the results showed that the two enriched signal pathways occurred in the eosinophils, neutrophils, macrophages, and T lymphocytes. In recent years, cytokine–cytokine receptor interaction has been considered a response to infectious agents and has been reported to be involved in the resolution of inflammation, which is linked to clinical mastitis [28]. A total of 39 DEGs participated in the Fc gamma R-mediated phagocytosis pathway, ranking fifth in the list of pathways. Phagocytosis is an essential element of the immune response permitting the elimination of pathogens, cellular debris, and apoptotic cells. This result suggests the importance of the recruitment and activation of macrophages and neutrophils in infectious sites during the later stage of *Staph*. *aureus* mastitis.

Many DEGs play important roles in host defense, inflammation, and tissue damage and healing. In a susceptible cow host, the persistence of bacteria pathogens, such as *Sta*. *aureus*, results in aberrant and extended inflammation and subsequent destruction of the mammary gland structures. Therefore, the 10 DEGs of the collagen family are important to heal the damaged tissue. For instance, Collagen Type I Alpha 1 (COL1A1) is up-regulated in the mastitic mammary gland and associated with tissue damage and repair during the later stages of infection [29]. Furthermore, the components of the complement system, such as C1S, C2, C3, C4A, C6, C7, and C8 genes, were differentially expressed; they underwent alternative splicing. The complement system is an essential component of the innate immune response, which is activated after molecular patterns associated with microorganisms, abnormal host cells, and modified molecules in the extracellular environment have been recognized. The consequent proteolytic cascade tags the complement activator for elimination and elicits a proinflammatory response, resulting in the recruitment and activation of immune cells from both the innate and adaptive immune systems [30]. These complement genes are good candidates for improving resistance to mastitis.

Several inflammation and immune-related genes were also highly expressed and alternatively spliced in the udder tissue. Duffy (Fy) antigen/receptor for chemokines (ACKR1, also named DARC) exhibited a 1.73-fold change and retained intron, which can bind to most inflammatory chemokines, activate and recruite leukocytes in the immune system [31]. Serin peptidase inhibitors SERPINA1 and SERPINA11 were highly expressed in the mammary glands, exhibiting 2.52-fold and 1.66-fold changes, respectively (Table B in S3 File). SERPINA1 encodes a serum protease inhibitor that can protect tissues against neutrophil attack and is associated with decreased bovine somatic cell score as well as increased milk yield, milk fat yield, and productive life [32]. Milk fat globule-EGF factor 8 (MFGE8) transcript, which has a 2.44-fold change, was highly expressed in HS3A (RPKM = 218) and HS8A (RPKM = 1183). Mfge8, a soluble milk glycoprotein, is known to regulate inflammation and immunity by mediating the clearance of apoptotic lymphocytes and epithelial cells [33]. The expression of Fc fragment of IgG, low affinity IIb, receptor (FCGR2A, also named CD32) in the mastitis-infected bovine tissue was up-regulated by 1.87-fold compared with that in the healthy tissue. CD32 can participate in biological processes, such as immune response, regulation of immune response, and anti-inflammation [34]. Programmed cell death 4 (neoplastic transformation inhibitor, PDCD4) encoding a tumor suppressor, a highly expressed transcript, was up-regulated in HS8A. PDCD4 is a proinflammatory protein that promotes the activation of the transcription factor NF-κB and suppresses interleukin 10 [35]. A 1.53-fold down-regulation of immunoglobulin lambda-like polypeptide 1 (IGLL1) was found in the mastitic tissue. Bovine IGLL1 is homologous to the variable and the constant domain of the immunoglobulin light chain, which is related to immune function, particularly for B cells and γδ T cells [36]. In the mammary gland, ENSBTAG00000031160 (IGLL1) was identified as a novel abundant transcript similar to immunoglobulin lambda-like polypeptide 1 precursor (immunoglobulin-related 14.1 protein), thereby warranting further functional investigation.

Bovine mastitis, which is an inflammation-driven disease of the mammary gland, occurs in response to physical damage or pathogen infection. *Staph*. *aureus* infection tends to cause mild, subclinical inflammation and persistent infection. Ineffective pathogen clearance frequently leads to chronic and persistent infection [37]. Clinical mastitis is a complex trait, and the different genes regulating immune responses are known to be pathogen specific [28]. The mounting of inflammation, defense, and activation of the innate immune system are complex processes involving many different genes, regulatory pathways, and networks, as well as a multitude of environmental factors, which all provide defense lines against pathogens. The complexity of this system possibly necessitates very different mechanisms that provide and shape effective, rapid, and reversible responses with limited resources [38]. AS, which is a versatile mechanism of gene expression regulation, is one of the most significant contributors to the functional complexity of the mammalian genome [8,20]. This process may indicate that different pathogens exploit different host mechanisms invasion strategies. In this study, we performed the first analysis of AS complexity in the mammary gland tissues of cows by combining RNA-Seq data and bioinformatics. We also identified 8,549 (52.62%) and 8,325 (51.24%) unigenes were alternatively spliced, exhibiting 34,523 and 30,410 AS events in the healthy and mastitic tissues, respectively. Many of these genes, which are immune-, defense-, and inflammation-related, underwent different and specific splicing patterns and events. For example, 12 healthy-group-specific genes (MYD88, C2, TFE3, VDAC1, ENPP2, TLR6, OAS2, IL27R, CD163, CD8A, ENPP1, and SAA3) and 8 mastitic-group-specific genes (NCF1, ADA, ITGB6, MAP2K3, MLF2, CSF3, POLR3C, and TNFRSF1B) retained introns. Bovine Myeloid Differentiation Primary Response 88 (MYD88), which retained intron 1 in the healthy group, is a critical protein in the lipopolysaccharide (LPS)-induced NF-κB and apoptotic signaling pathways. This protein plays functional roles in transducing LPS signaling from TLR4 to downstream effector molecules involved in NF-κB activation in endothelial cells [39]. Integrin Subunit Beta 6 (ITGB6), which retained intron 15 in the mastitic tissue, was found to be differentially expressed in mastitis-resistant and susceptible sheep infected with *Staph*. *epidermidis* [40]. The predicted protein structure of the novel transcript ITGB6 was altered because of intron retention; as a result, the function of ITGB6 was affected and risk of mastitis was increased. TNFRSF1B, which retained intron 8, was assigned to the inflammatory response and immune response GO terms. ADA was not differentially expressed in the two groups. However, ADA retained intron 5 and deleted exon 2 in the mastitic group. The deficiency of ADA causes a severe combined immunodeficiency disease, in which the dysfunctions of both B and T lymphocytes occur with impaired cellular immunity and decreased production of immunoglobulin (http://www.genecards.org/). We speculate that the domain changes in ADA would alter its function via the AS mechanism, thus leading to different susceptibilities to mastitis.

In our recent studies, we have reported that the novel transcripts of genes produced by AS are significant in bovine mastitis resistance and susceptibility [15,16,41,42]. Among these DEGs, genes that participated in the immune, defense, and inflammatory responses were identified by DAVID clustering to show numerous AS events. In this study, several genes previously reported to initiate antimicrobial and antiviral immune responses, such as C-C Motif Chemokine Ligand 5 (CCL5) [43,44], Colec2 [45], Lactotransferrin (LTF) [46–48], CD46 Molecule (CD46) [16], and Neutrophil Cytosolic Factor 1 (NCF1) [42], showed specific AS events in mastitis-infected mammary glands. Among these genes, CCL5 and CSN1S2 were included in the top 100 highly expressed genes in the HS8A and HS3A mammary glands. We established in our previous study that LTF has two splice variants associated with bovine *Sta*. *aureus* mastitis [46]. The BOLA-DQA2-SV1 transcript, which belongs to the BOLA class II genes, plays an important role in mastitis resistance among dairy cattle [41]. In the current study, CD46 exhibited four splicing patterns, namely, exon skipping, intro retention, and alternative 5′ and 3′ splicing. We showed in our previous study that CD46 probably plays a critical role in the *Strepotococcus* mastitis risk among dairy cows via an alternative splicing mechanism caused by a functional mutation in intron 8 [16]. Two splice variants of NCF1 were sharply up-regulated in the mammary tissues, blood, and neutrophils of mastitis-infected cows compared with those of healthy cows. Moreover, a splicing-related SNP was associated with increased milk somatic cell score in cows [42].

Several QTLs have been found to affect resistance to clinical mastitis in dairy cattle populations [23–26]. We also found many novel transcripts, some of which were located in the known QTLs associated with clinical mastitis. For example, the novel 251 bp TU98975 was identified at Chr8:61,371,653–61,371,903 in the mastitic group. This region of chromosome 8, from 61,042,106 bp to 61,507,067 bp, has been found to be related to clinical mastitis in US Holstein cattle population. This chromosome can be used to explain the 10 highest proportions of variance; however, no annotated genes were found in this region [28]. Our finding indicates that the novel transcript TU98975 may be a candidate gene for mastitis resistance, providing a novel explanation to the finding of the previous study.

## Conclusion

We infer that gene-specific AS events in healthy bovine mammary glands may have positive effects on immunoregulatory activity by protecting the host from infection. By contrast, mastitic-specific AS events have negative influences on resistance, thereby increasing the susceptibility to mastitis. Therefore, our findings provide a valuable basis for further understanding of the molecular mechanism underlying mastitis resistance and susceptibility in dairy cows. The diverse and specific splicing patterns and events of genes lead to the loss-of-function and gain-of-function of genes and diversity and contribute to the potential complex genetic resistance against mastitis in dairy cows.

## Supporting Information

S1 File **Fig A.** Sequencing randomness assessment and distribution statistics of reads mapped onto the reference gene. **Fig B.** Schematic diagram of seven kinds of alternative splicing. **Fig C.** Sketches of the algorithms of the four splicing patterns. **Fig D.** Schematic diagram of the gene structural optimization method.
(DOCX)

S2 File **Table A.** A total of 1352 DEGs including 602 up-regulated genes and 750 down-regulated genes were identified in the healthy and mastitic groups. **Table B.** Top 100 highly expressed genes in HS8A and HS3A mammary gland tissues.
(XLSX)

S3 File **Table A.** DEGs enriched in molecular function category. **Table B.** DEGs enriched in biological process category. **Table C.** DEGs enriched in cellular component category.
(XLSX)

S4 File **Table A.** A total of 124 DEGs participated in immune system process on the basis of GO term. **Table B.** A total of 74 DEGs participated in defense response on the basis of GO term. **Table C.** A total of 30 DEGs participated in immune response on the basis of GO term. **Table D.** A total of 22 DEGs participated in inflammatory response on the basis of GO term. **Table E.** List of genes participated in immune, inflammation and defense and expressed with above 1.5-fold change in two groups.
(XLSX)

S5 File **Table A.** Novel transcripts harboring QTLs associated with clinical mastitis in the healthy mammary gland tissue. **Table B.** Novel transcripts harboring QTLs associated with clinical mastitis in the mastitic mammary gland tissue.
(XLSX)

S6 File **Table A.** Genes exhibited retained intron patterns in HS3A and HS8A and participated in immune, defense and inflammatory responses. **Table B.** Overlapped genes with the same and different exon skipping pattern expressed in HS8A and HS3A and participated in immune, defense and inflammatory responses. **Table C.** Genes with 5’-splicing events were healthy-group-specific and mastitic- group-specific and participated in immune, defense and inflammatory responses. **Table D.** Alternative 5' splicing site pattern of genes expressed and participated in immune, defense and inflammatory responses in healthy and mastitis groups. **Table E.** Genes with 3’-splicing events were healthy-group-specific and mastitic- group-specific and participated in immune, defense and inflammatory responses. **Table F.** Alternative 3' splicing site pattern of genes expressed and participated in immune, defense and inflammatory responses in healthy and mastitis groups.
(XLSX)
