# Correction: Deciphering Transcriptome and Complex Alternative Splicing Transcripts in Mammary Gland Tissues from Cows Naturally Infected with *Staphylococcus aureus* Mastitis

**DOI:** 10.1371/journal.pone.0167666

**Published:** 2016-12-01

**Authors:** Xiu Ge Wang, Zhi Hua Ju, Ming Hai Hou, Qiang Jiang, Chun Hong Yang, Yan Zhang, Yan Sun, Rong Ling Li, Chang Fa Wang, Ji Feng Zhong, Jin Ming Huang

The images for Figs 2 and 3 are incorrectly switched. The image that appears as Fig 2 should be Fig 3 and the image that appears as Fig 3 should be Fig 2. The figure captions appear in the correct order.

**Fig 2:**
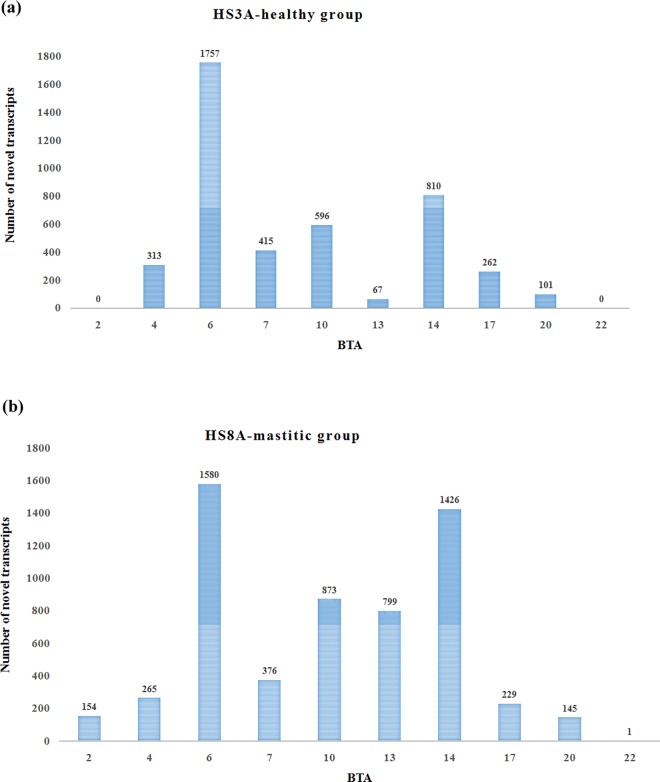
Novel transcripts harboring QTLs associated with clinical mastitis in the two groups. BTA: Bovine autosome.

**Fig 3:**
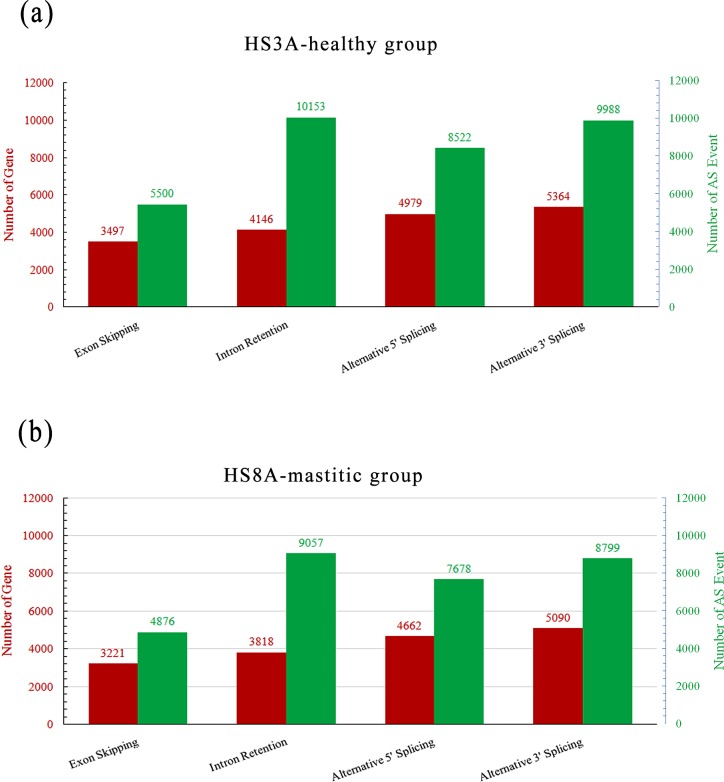
Genes and their patterns of alternative splicing events in healthy and mastitic cow’s mammary gland tissue. The red and green histograms show the numbers of genes and the corresponding numbers of alternative splicing events, respectively.
