# Supplementary material for: Deciphering Transcriptome and Complex Alternative Splicing Transcripts in Mammary Gland Tissues from Cows Naturally Infected with Staphylococcus aureus Mastitis
Source: PLoS One. 2016 Jul 26;11(7):e0159719. doi: 10.1371/journal.pone.0159719 (PMC4961362; doi:10.1371/journal.pone.0159719)
Supplement: S1 File — Fig A. Sequencing randomness assessment and distribution statistics of reads mapped onto the reference gene. Fig B. Schematic diagram of seven kinds of alternative splicing. Fig C. Sketches of the algorithms of the four splicing patterns. Fig D. Schematic diagram of the gene structural optimization method. (DOCX) [file pone.0159719.s001.docx]

**S1 File:**


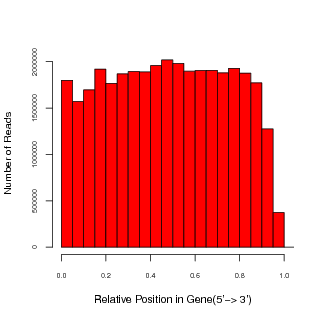
 **(1) (2)**


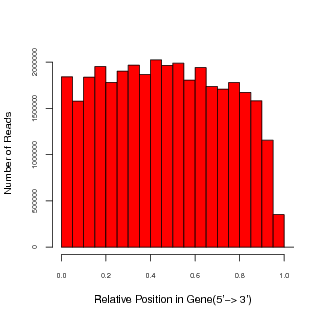


**Figure A. Sequencing randomness assessment and distribution statistics of reads mapped onto the reference gene.** (1) HS3A library (Healthy cows). (2) HS8A library (Mastitic cows). The relative position in gene corresponds to the ratio of reads in the reference genes to the length of the reference genes.


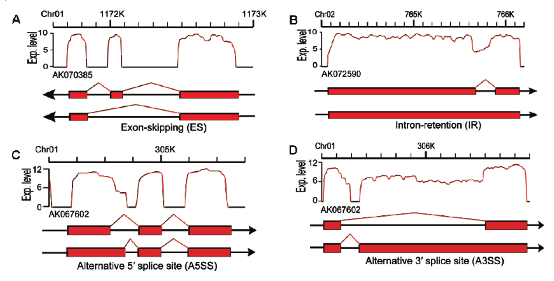


(3)

(1)

(2)

(4)

(1)


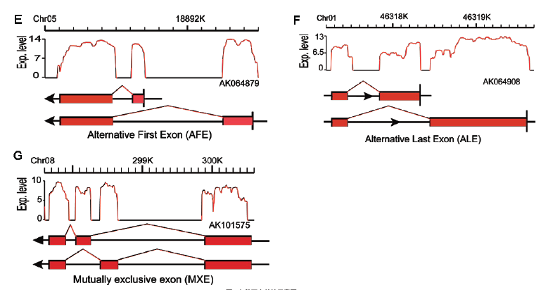


(7)

(6)

(5)

**Figure B. Schematic diagram of seven kinds of alternative splicing.** Expression level is equal to the value of log_2_(Reads). The splicing patterns of (5), (6), and (7) are not provided in this study because of their low detection reliability in the SOAPsplice program.

(1)Exon skipping

(2) Exon skipping

(3) Intron retention

(4) Alternative 5′ splice site

(5) Alternative 3′ splice site

**Figure C. Sketches of the algorithms of the four splicing patterns.** (1) If transcripts 1 and 2 have junction sites, exon skipping occurs in both transcripts 1 and 2. (2) Exon skipping occurs in both transcripts 1 and 2. (3) An intron retention event occurs between exons 1 and 2 when the following five conditions are met: ① junction 1 is detected, which indicates an intron between exons 1 and 2; ② 90% of this intron is covered by unique mapping reads; ③ the coverage depth of the intron is at least 15% of the coverage depth of exon 1 or 2; ④ the 5 bp upstream and downstream portions of both boundaries of the intron should be covered by the reads; ⑤ the intron region cannot be covered by other another gene. (4) If either junction 2 or 3 has the same 3′ splice site as but different 5′ splice site from those of junction 1 and junction 1 is detected, then an alternative 5′ splicing event occurs between exons 1 and 2. (5) If either junction 2 or 3 has the same 5′ splice site as but different 3′ splice site from those of junction 1 and junction 1 is detected, then an alternative 3′ splicing event occurs between exons 1 and 2.


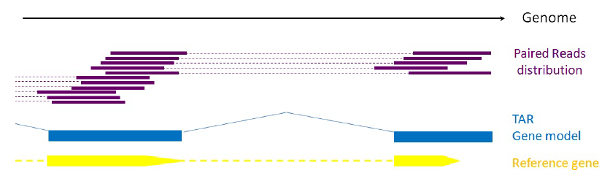


**Figure D. Schematic diagram of the gene structural optimization method.**
